# Further observations on *LKB1/STK11* status and cancer risk in Peutz–Jeghers syndrome

**DOI:** 10.1038/sj.bjc.6601030

**Published:** 2003-07-15

**Authors:** W Lim, N Hearle, B Shah, V Murday, S V Hodgson, A Lucassen, D Eccles, I Talbot, K Neale, A G Lim, J O'Donohue, A Donaldson, R C Macdonald, I D Young, M H Robinson, P W R Lee, B J Stoodley, I Tomlinson, D Alderson, A G Holbrook, S Vyas, E T Swarbrick, A A M Lewis, R K S Phillips, R S Houlston

**Affiliations:** 1 Section of Cancer Genetics, Institute of Cancer Research, Sutton, Surrey SM2 5NG, UK; 2 Department of Genetics, St. Georges' Hospital, London SW17 0QT, UK; 3 Department of Clinical Genetics, Guy's Hospital, London SE1 9RT, UK; 4 Wessex Clinical Genetics Service, The Princess Anne Hospital, Southampton SO16 5YA, UK; 5 Academic Department of Pathology, St Mark's Hospital, Watford Road, Harrow HA1 3UJ, UK; 6 Polyposis Registry, St Mark's Hospital, Watford Road, Harrow HA1 3UJ, UK; 7 Department of Gastroenterology, Epsom General Hospital, Surrey, KT18 7EG, UK; 8 Department of Gastroenterology, University Hospital Lewisham, London SE13 6LH, UK; 9 Department of Clinical Genetics, St Michael's Hospital, Bristol BS2 8EG, UK; 10 Department of Surgery, Huddersfield Royal Infirmary, Huddersfield HD3 3EA, UK; 11 Department of Genetics, City Hospital, Nottingham NG5 1PB, UK; 12 Department of Surgery, City Hospital, Nottingham NG5 1PB, UK; 13 Department of Surgery, Hull Royal Infirmary, Hull HU3 2JZ, UK; 14 Department of Surgery, Eastbourne Hospital, East Sussex BN21 2UD, UK; 15 Molecular and Population Genetics Laboratory, Imperial Cancer Research Fund, London WC2A 3PX, UK; 16 Department of Surgery, Bristol Royal Infirmary, Bristol BS2 8HW, UK; 17 Department of Surgery, Royal United Hospital, Bath BA1 3NG, UK; 18 Department of Medicine, Salisbury District Hospital, Salisbury SP2 8BJ, UK; 19 Department of Gastroenterology, New Cross Hospital, Wolverhampton WV10 0QB, UK; 20 Department of Surgery, Royal Free Hospital, London NW3 2QG, UK

**Keywords:** Peutz–Jeghers syndrome, *LKB1/STK11*, mutation, cancer risk

## Abstract

Germline mutations in the *LKB1/STK11* tumour suppressor gene cause Peutz–Jeghers syndrome (PJS), a rare dominant disorder. In addition to typical hamartomatous gastrointestinal polyps and pigmented perioral lesions, PJS is associated with an increased risk of tumours at multiple sites. Follow-up information on carriers is limited and genetic heterogeneity makes counselling and management in PJS difficult. Here we report the analysis of the *LKB1/STK11* locus in a series of 33 PJS families, and estimation of cancer risks in carriers and noncarriers. Germline mutations of *LKB1/STK11* were identified in 52% of cases. This observation reinforces the hypothesis of a second PJS locus. In carriers of *LKB1/STK11* mutations, the risk of cancer was markedly elevated. The risk of developing any cancer in carriers by age 65 years was 47% (95% CI: 27–73%) with elevated risks of both gastrointestinal and breast cancer. PJS with germline mutations in *LKB1/STK11* are at a very high relative and absolute risk of multiple gastrointestinal and nongastrointestinal cancers. To obtain precise estimates of risk associated with PJS requires further studies of genotype–phenotype especially with respect to *LKB1/STK11* negative cases, as this group is likely to be heterogeneous.

Peutz–Jeghers syndrome (PJS; MIM 175200) is an autosomal dominant disorder characterised by a specific form of hamartomatous polyposis of the gastrointestinal tract, and by melanin pigmentation of the lips, perioral region and buccal mucosa, fingers and toes, and other sites (Tomlinson and Houlston, 1997). Approximately three-quarters of PJS are familial, the remainder resulting from new mutations or low-penetrance variants. PJS typically presents in early childhood with pigmentation or with complications of small bowel polyps–intussusception, obstruction or bleeding.

Although PJS polyps are seen most commonly in the small bowel, they can occur throughout the gastrointestinal tract (Tomlinson and Houlston, 1997) and at other extra-intestinal sites such as the kidney, ureter, gall bladder, bronchus and nasal passage (Westerman *et al*, 1999; Sommerhaug *et al*, 1970; Wada *et al*, 1987). The polyps seen in PJS have a muscular core and are generally classified as being hamartomas. Nevertheless, adenomatous change may occur in polyps and they may become malignant, and an increased risk of jejunal and other small bowel tumours is recognised (Gruber *et al*, 1998).

In addition to an elevated risk of gastrointestinal malignancies, an increased risk of cancers at other sites is recognized; in particular, breast, pancreas, ovary, uterus, cervix, lung and testicular cancers have been reported (Giardello *et al*, 1987,^2000^; Spigelman *et al*, 1989). Testicular sex cord and Sertoli-cell tumours may occur in prepubertal boys affected with PJS leading to sexual precocity and gynaecomastia (Wilson *et al*, 1986; Coen *et al*, 1991; Young *et al*, 1995). The production of oestrogen in ovarian tumours in girls with PJS has also been reported causing isosexual precocity (Sohl *et al*, 1983).

Germline mutations in the serine/threonine kinase gene (*LKB1/STK11)* on chromosome 19p13.3 have been shown to cause PJS (Hemminki *et al*, 1997; Hemminki *et al*, 1998; Jenne *et al*, 1998). This gene has a putative coding region of ∼1.3 kb, composed of nine exons, and functions as a tumour suppressor.

Previous studies have shown that between 30 and 82% of patients have no detectable germline mutations in *LKB1/STK11* (Mehenni *et al*, 1998; Nakagawa *et al*, 1998; Jiang *et al*, 1999; Wang *et al*., 1999; Westerman *et al*, 1999b; Ylikorkala *et al*, 1999; Boardman *et al*, 2000; Yoon *et al*, 2000; Olschwang *et al*, 2001). Families with PJS unlinked to 19p13.3 have also been reported, suggesting that the disease is heterogeneous (Jiang *et al*, 1999; Westerman *et al*, 1999b; Yoon *et al*, 2000). Furthermore, a second PJS locus on chromosome 19q13.4 has been proposed on the basis of linkage in one family (Mehenni *et al*, 1998).

The clinical features of PJS are variable especially with respect to cancer risks. It is likely that inter- and intrafamilial differences in disease expression reflect in part the influence of different germline mutations.

To further our knowledge about the relation between genotype and cancer risk in PJS, we have related disease expression to *LKB1/STK11* status in 33 families.

## PATIENTS AND METHODS

### Patients

Thirty-three index patients with PJS were ascertained through colorectal surgeons, gastroenterologists and geneticists within the UK. Clinical information was collected on all patients using a standard proforma and through access to patients' medical records. PJS was defined according to published diagnostic criteria (Giardello *et al*, 1987)–histopathologically verified hamartomatous polyps with at least two of the following: small bowel polyposis, mucocutaneous melanotic pigmentation and family history of the disease. Patients were asked to provide details of any cancer in first- and second-degree relatives. There was no selection of cases for a family history of cancer. Clinical information and samples were obtained with informed consent and Local Ethical Review Board approval in accordance with the tenets of the Declaration of Helsinki.

### Mutational analysis of *LKB1/STK11*

Genomic DNA from PJS patients was isolated from EDTA venous blood samples using a standard sucrose lysis protocol. The search for germline mutations in *LKB1/STK11* was performed using conformational sensitive gel electrophoresis (CSGE) as described by Ganguly *et al* (1993). Published oligonucleotide primer sequences were used to amplify each of the nine exons of *LKB1/STK11* (Bignell *et al*, 1998). Any fragments showing migration shifts were reamplified and sequenced directly using the ABI Prism dRhodamine Terminator Cycle Sequencing Ready Reaction Kit and an ABI377 Genetic Analyser. For all samples with possible mutations, sequencing was replicated in forward and reverse orientation using an additional affected family member (or using the original patient if no other affected individual had been sampled) in order to confirm the presence of the mutation. A search for large-scale deletions in *LKB1/STK11* was made by long-range PCR. Amplification of exons 3–8 of *LKB1/STK11* was undertaken using the Expand Long Template PCR System (Roche Diagnostics, UK).

Nucleotide changes identified were coded according to the published sequence of *LKB1/STK11* (Genbank accession numbers: exon 1, AF032984; exons 2–8, AF032985; exon 9, AF032986) and referenced to the Human Gene Mutation Database (http://archive.uwcm.ac.uk/uwcm /mg).

A search of the literature was made using the electronic database MEDLINE (National Library of Medicine, USA) for additional mutations reported to be associated with PJS which were not referenced in the Human Gene Mutation Database. *LKB1/STK11* protein sequences of *Homo sapiens* (GenBank accession number NP 000446), *Mus musculus* (NP 035622) and *XEEK1* (Q91604) were obtained from the NCBI protein database. They were aligned using Clustal W (1.82) multiple sequence alignment program (http://www.ebi.ac.uk/clustalw/).

### Statistical analyses

Statistical analyses were performed using the statistical software program STATA Version 6 (Stata Corporation, TX, USA. http://www.stata.com). The 95% confidence interval (CI) of the estimate of the frequency of *LKB1/STK11* mutations in PJS was estimated from the binomial distribution. The association between categorical variables was made using either Fisher or *χ*^2^ tests, and differences in the distribution of continuous variables were evaluated using the Mann–Whitney *U*-test. Estimation of cancer risks was made excluding cases that had developed neoplasms either before or at the time of presentation of PJS. Estimates of cancer risks were obtained from survival analyses and standardised mortality ratios (SMRs). SMRs for cancers were determined using life-table methods. Cases were considered at risk from age 5 until the date of diagnosis of cancer or date of ascertainment, censoring at age 65. Expected numbers of cancers were computed using age-, sex- and calendar period-specific mortality rates for England and Wales referenced to the International Classification of Diseases, ninth revision (ICD-9)–all cancers 140–208, cancers of the digestive organs and peritoneum 150–159 and breast carcinoma 174. Two-sided 95% CIs for relative risk estimates are based on the Poisson distribution. A *P*-value of 0.05 was considered statistically significant.

## RESULTS

Table 1

**Table 1:** Family history, clinical characteristics and *LKB1/STK11* status of index patients

				***LKB1/STK11* mutation**			**Clinical features**				**Cancer**	
**Patient**	**Sex**	**Age**	**Familial/sporadic**	**Exon**	**Mutation**	**Effect of mutation**	**Age at diagnosis**	**Polyp site in GI tract**	**Intussusception at age (years)**	**Extra-intestinal polyps**	**Index case**	**Relative**
PJ77	M	23	F	1	208G>T	E70X	13	SB	14	—	—	Mother cervix
												Aunt colorectal
PJ48	M	37	S	1	180C>A	Y60X	2	SB, LB	2, 7, 17, 24	—	—	—
PJ36	F	26	S	2	335–337del	Q112fsX17	7	ST,SB,LB	8, 9,15,20	—	—	—
PJ60	F	61	S	2	368–370del	Q123fsX6	16	SB, LB	16, 25, 55	—	Breast	Grandfather stomach
PJ52	F	34	F	3	454C>T	Q152X	18	SB, LB	18	—	—	—
PJ56	F	40	F	3	426–428del	V143fsX144	8	SB, LB	8, 18	Vocal cords	—	Mother stomach
PJ59	F	30	F	4	470T<C	F157S	4	SB, LB	7, 14 ,27	—	—	—
PJ20	F	31	F	4	580G>A	D194N	15	SB	15	—	—	Grandfather stomach
PJ69	F	31	S	IVS5	IVS5+1G>A	?altered splicing	18	SB, LB	21, 24	—	—	—
PJ24	F	48	S	5	718–719insA	S240fsX26	20	SB	20, 33	—	—	—
PJ45	F	31	F	5	725G>A	G242E	10	ST, SB	4, 19, 26	—	—	Father pancreas
PJ47	F	31	F	6	815–816insA	Y272X	25	ST, SB, LB	25, 29	—	—	—
PJ51	M	38	F	6	842–844del	L282fsX5	4	ST, SB, LB	13, 14, 15,16,18,22,23,33,35,36	—	—	—
PJ42	F	35	F	6	842–844del	L282fsX5	10	SB, LB	30	—	—	Mother breast
PJ35	F	39	F	7	910C>T	R304W	16	ST,SB	16	—	Breast	Mother, grandmother breast
PJ33	M	61	S	IVS8	IVS8-2A>G	?altered splicing	31	LB	—	—	—	—
PJ61	M	39	S	IVS8	IVS8-2A>G	?altered splicing	19	ST, SB, LB	23, 32	—	—	—
PJ62	F	65	F		ND	—	13	SB, LB	13, 40	Nasal	—	—
PJ25	M	56	S		ND	—	20	SB, LB	20	—	—	—
PJ40	M	39	S		ND	—	26	SB, LB	28	—	—	—
PJ01	F	39	S		ND	—	5	SB	19	—	—	—
PJ39	M	21	S		ND	—	11	SB	11, 20	—	—	—
PJ49	F	54	F		ND	—	22	ST, SB, LB	22 , 52	—	—	—
PJ55	M	41	S		ND	—	15	ST, SB, LB	15, 20, 34	—	—	—
PJ66	F	22	S		ND	—	5	ST, SB, LB	9, 20	Ear and nasal	—	—
PJ67	M	35	F		ND	—	2	ST, SB, LB	2,12,13,14,18	—	—	Mother stomach
PJ70	F	37	S		ND	—	3	ST, SB, LB	—	—	—	Aunt breast
PJ37	F	40	S		ND	—	38		—	—	—	—
PJ64	M	50	S		ND	—	5	ST, LB	7, 14, 26	Pharyngeal	—	—
PJ68	M	44	S		ND	—	23	ST, SB, LB	23	—	—	Mother oesophagus
PJ100	F	39	S		ND		27	LB	—	—	—	—
PJ102	M	15	S		ND		6	LB	—	—	Sertoli cell	—
PJ71	M	39	S		ND	—	34	SB	—	—	—	—

ST=stomach; SB=small bowel; LB=large bowel. ND=none detected. Positions refer to the *LKB1* cDNA sequence (Genbank U63333).

details the clinical characteristics and family histories of the 33 index patients analysed. Of these cases, 13 were familial and 20 sporadic. Germline *LKB1/STK11* mutations were identified in 17 of the 33 (52%; 95% CI: 33–69%) patients (Table 1), in exons 1–8.

We cannot exclude the possibility that some mutations may have gone undetected; however, under test conditions, we have found that CSGE can detect all small insertions and deletions and ∼90% of single-base substitutions. In addition, we have examined for the possibility that some cases might harbour large-scale deletions in *LKB1/STK11*. It is therefore unlikely that we have failed to detect coding mutations, and, allowing for 90% sensitivity, the results suggest that the mutations in *LKB1/STK11* account for at best 75% of PJS cases (the upper 95% confidence limit). Two patients carried the same mutation in exon 6 (PJ42 and PJ51) and two carried the same mutation at the 5′ splice site of exon 8 (PJ33 and PJ61). These four patients were ascertained from different centres and were not known to have any common ancestry. Nevertheless, as all are from the UK, it is probable that these mutations have a common origin, although identical *LKB1/STK11* mutations without evidence of common ancestry have been reported (Hemminki *et al*, 1998; Resta *et al*, 1998; Wang *et al*, 1999; Westerman *et al*, 1999b; Ylikorkala *et al*, 1999). None of the patients studied were shown to harbour large-scale deletions of *LKB1/STK11*.

No significant bias towards mutations in exons 1 or 6 was observed, but no exon 9 mutations were identified. Seven of the 15 different mutations identified have not been reported previously–336delG (Q112fsX17), 369delG (Q123fsX6), 427delG (V143fsX144), 718_719insA (S240fsX26), G725A (G242E), 815_816insA (Y272X), IVS8-2A>G (altered splicing). In all, 11 of the mutations are predicted to lead to a truncated protein (four nonsense mutations, four frameshift deletions, one frameshift insertion and two splice site mutations). The other mutations identified were missense mutations, three of which have previously been reported to be pathogenic (Resta *et al*, 1998; Westerman *et al*, 1999b; Ylikorkala *et al*, 1999). All are nonconservative amino-acid changes that are highly conserved among human, mouse and *Xenopus* homologues of *LKB1/STK11* and reside within the protein kinase core of *LKB1/STK11* (Collins *et al*, 2000).

Table 2

**Table 2:** Location of *LKB1/STK11* mutations in PJS patients in this study and published reports. Also shown are the cancers associated with mutations

**Reference**	**Exon 1**		**Exon 2**		**Exon 3**		**Exon 4**		**Exon 5**		**Exon 6**		**Exon 7**		**Exon 8**	**Exon 9**
This study^a^	E70X^cx,co^		112fs		Q152X		F157S		240fs	IVS5+1	Y272X		K296X	IVS7-2		
	Y60X		123fs^b^		143fs^st^		D194N		G242E^p^	G>A	282fs^b^		E291V^se^	A>G		
													R304W^b^			
i^a^	E57X		ex2_ex3								277fs		302del5		ex8 del	
	65fs		del													
	E70X															
	L67P															
	Y60X															
	55fs															
	K84X															
																
ii^a^						**IVS3-1**	**156_307**		**240fs**		Y252X					
						**G>A**	**invdel**				280fs					
																
Iii									**Q220X**							
																
iv	**38fs**	**IVS1-1**			**139fs**				**244fs**	**IVS5+2**	**Y246X**					
		**G>C**								**insT**	**B247del**					
											280fsX6					
											280fsX4					
																
v^a^	52fs				Q152X		F157S		201fs	IVS5+3	263fs^p^		304fs^p, sb^	IVS7-1	W308X	
	K84X^b,cx, mm^				137del4		N181Y		Q220X	A>T	257fs^co^			G>C^co^		
	R86X										248fs^o, th^					
											280fsX6					
																
vi							175del2				262fs					
											G251S		R304W			
																
vii^a^	50del4						D176N			IVS5+5		IVS6	301fs		W308C	
							191fs			G>A		del				
viii^a^											844insC ^co, p, pr^	IVS6+3	R297S^co, ut^		971del6	
												G>C	H272Y^co, th^			
																
ix	54fs	IVS1-2	Q100X				D162N						R297K		331fs	
	51fs	A>G					G163D									
		IVS1-1					L164M									
		G>C					D194N									
																
x^a^	L67R	IVS1+	Y118X		M136R	IVS3-2	L182P	IVS4-2	G242V	IVS5+1	263fs		303del3		319fs	
	Y60X ^b^	1	117fs		C132X^b, d^	A>G ^p^		A>T	222fs	G>A	251fs					
	57fs	G>A							212fs		281fs ^p, k^					
	52del ^k^								G242W		248fs					
																
xi	Y60X		K108R				176fs				281fs					K416X^ut^
			108del2													
																
xii^a^			ex2-7del ^d^													
																
xiii		IVS1-2							S232P		E256A			P324L		
		A>C												342fs		
																
xiv	41fs ^se^						172fs ^p, o^		Q220X							
							157fs ^b^									
xv	37fs			ex2 del			188fs		213fs							
									221fs							
									262fs							
																
xvi^a^		IVS1-2					Q170X									
		A>G ^te^														

a search for large-scale deletions made.

Mutation changes are described at the protein level.

Key to cancers: co=colon; p=pancreas; pr=prostate; b=breast; cx=cervix; se=sertoli cell; ut=uterus; o=ovary; k=kidney; d=duodenum; te=testis.

References: i, Hemminki *et al* (1998); ii, Jenne *et al* (1998); iii, Gruber *et al* (1998); iv, Nakagawa *et al* (1998); v, Ylikorkala *et al* (1999); vi, Resta *et al* (1998); vii, Mehenni *et al* (1998); viii, Boardman *et al* (2000); xi, Westerman *et al* (1999); x, Olschwang *et al* (2001); xi, Wang *et al* (1999); xii, Jiang *et al* (1999); xiii, Yoon *et al* (2000); xiv, Kruse *et al* (1999); xv, Miyaki *et al* (2000); xvi, Abed *et al* (2001).

shows the positions of the mutations observed in our study and in previously published reports (Hemminki *et al*, 1998; Gruber *et al*, 1989; Jenne *et al*, 1998; Mehenni *et al*, 1998; Nakagawa *et al*, 1998; Resta *et al*, 1998; Jiang *et al*, 1999; Kruse *et al*, 1999; Wang *et al*, 1999; Westerman *et al*, 1999b; Ylikorkala *et al*, 1999; Boardman *et al*, 2000; Miyaki *et al*, 2000; Yoon *et al*, 2000; Olschwang *et al*, 2001; Abed *et al*, 2001). Overall, most mutations reported to date have been frameshift or nonsense mutations and thus result in a truncated protein. In-frame deletions or missense mutations appear to occur less frequently generally at conserved amino acids in the kinase core.

Very few cases of PJS appear to be the consequence of large-scale deletions of *LKB1/STK11*; however, not all studies have systematically searched for such genetic changes (Table 2).

Disease expression in PJS is well documented to display inter- and intrafamilial variation (Burdick *et al*, 1982; Foley *et al*, 1998). Establishing a relationship between a number of the features of the disease and genotype is, however, inherently problematic because features typical of the disease are criteria for ascertainment. Nevertheless, there was no evidence that the ages at diagnosis are significantly different in carriers and noncarriers–mean ages of index cases, 13.9 y and 13.6 years, respectively. Furthermore, the distribution of polyps and rates of laparotomy were not significantly different between the groups.

Some previously reported studies have reported no association between detectable *LKB1/STK11* mutation and family history (Hemminki *et al*, 1998; Wang *et al*, 1999; Ylikorkala *et al*, 1999). In our study, 13 of the 33 index cases had a family history of PJS (39%). Of these 10 were carriers of mutations in *LKB1/STK11* (77%), but only seven (35%) patients with sporadic disease had mutations in *LKB1/STK11*. The higher prevalence of *LKB1/STK11* mutations in PJS patients with a family history of the disease compared with sporadic cases is statistically significant (*P*=0.03).

Extra-gastrointestinal polyps are a recognised feature of PJS. Four of the patients in our study had extra-intestinal polyps: one of these harboured an *LKB1/STK11* mutation and three did not.

Two patients had developed breast cancer since the diagnosis of PJS had been made–at ages 52 and 35 years. Both are carriers of an *LKB1/STK11* mutation. In addition, one patient had presented at age 6 with a Sertoli–Leydig cell stromal tumour. He did not harbour an, *LKB1/STK11* mutation. A high frequency of cancer was seen in the relatives of the familial cases–stomach (*n*=2, ages 32, 33 years), breast (*n*=2, ages 39, 51 years), colorectal (*n*=2, ages 43, 67 years), pancreas (*n*=1; age 50 years) and adenoma malignum of the cervix (*n*=1, age 43 years). All but the one case of stomach cancer was associated with *LKB1/STK11* mutations. Excluding the case presenting with a Sertoli–Leydig cell tumour, the index cases and their relatives provided a total of 70 individuals with PJS from which cancer risks could be estimated. These individuals provided a total of 2120 years at risk.

The probability of developing cancer by age 65 years in all PJS patients was 37% (95% CI: 21–61%). The observation of seven cancer deaths, four from gastrointestinal disease, between ages 5 and 65 years, equates to the SMR for all cancer of 9.9 (95% CI: 0.4–20.4; *P*<0.001) and for gastrointestinal cancer of 24.8 (95% CI: 0.7–63.6; *P*<0.001). Confining the analysis to *LKB1/STK11* mutation carriers, the probability of developing cancer by age 65 is 47% (95% CI: 27–73%), SMR of all and gastrointestinal cancers of 13.2 (95% CI: 0.5–27.1, *P*<0.001) and 32.0 (95% CI: 0.5–81.8, *P*<0.001), respectively. The risk of breast cancer in carriers was markedly increased, 29% by age 65 (95% CI: 12–62%); SMR, 13.9 (95% CI: 0.2–50.3, *P*<0.001).

## DISCUSSION

It is now well recognised that cancer risks are markedly elevated in PJS (Giardello *et al*, 1987,^2000^; Spigelman *et al*, 1989). Diagnosing PJS in the absence of mutation data, especially in those without a prior family history of the disease, can however be difficult as pigmentation may not always be present or can fade and polyposis is not always an invariable feature. Moreover, there is substantial phenotypic overlap with other syndromes such as Carney complex (Stratakis *et al*, 1998).

Over 75% of *LKB1/STK11* mutations reported have been frameshift or nonsense mutations and thus result in a truncated protein (Hemminki *et al*, 1998; Jenne *et al*, 1998; Mehenni *et al*, 1998; Nakagawa *et al*, 1998; Resta *et al*, 1998; Jiang *et al*, 1999; Kruse *et al*, 1999; Wang *et al*, 1999; Westerman *et al*, 1999b; Ylikorkala *et al*, 1999; Boardman *et al*, 2000; Miyaki *et al*, 2000; Yoon *et al*, 2000; Olschwang *et al*, 2001). In-frame deletions or missense mutations appear to occur less commonly at conserved amino acids within the kinase core of the expressed protein. Mutations reported to date have been scattered across exons 1–8. The distribution of mutations within the protein kinase core encoding region of *LKB1/STK11* does not appear to be random (*P*<0.05) and exons 1 and 6 appear to be preferentially involved accounting for ∼38% of all reported mutations. Only one mutation has been described in exon 9 (Wang *et al*, 1999)–a nonsense mutation removing 56 residues from the protein of 434 amino acids and as such resides outside the protein kinase core. Although the case was familial, other members of the family were not evaluated and hence the pathological significance of this mutation is questionable.

Our study showed that the risk for cancer, gastrointestinal and breast, associated with germline *LKB1/STK11* mutations is high and supports recent implementation of screening protocols suggested for patients (Wirtzfeld *et al*, 2001). In contrast to a number of other inherited cancer syndromes, cancer risks associated with germline *LKB1/STK11* mutations cancer risks are not so site specific. *LKB1/STK11* functions as a tumour suppressor in hamartomous polyps and in neoplasms. Some neoplasms develop from hamartomas; however, as *LKB1/STK11* has a role in a number of pathways involved in control of cell growth, it is likely that some mutations may confer an increased cancer risk through alternative mechanisms.

In our study, cancers were found in association with mutations in most exons. From studies published so far, there does not seem to be a specifically higher prevalence of any cancer associated with mutations in specific exons (Figure 1). However, one of the mutations we detected, R304W, appeared to be associated with a high risk of breast cancer. It is highly conceivable that certain mutations may be associated with higher risks of cancer at certain sites, as seen with *BRCA2* (The Breast Cancer Linkage Consortium 1999; Murphy *et al*, 2002). To formally assess such relationships will require a large number of observations.

Since Hemminki *et al* (1998) first showed that germline mutations in *LKB1/STK11* cause PJS, a number of studies have examined the prevalence of mutations in the syndrome. In our study, we identified the *LKB1/STK11* mutation in 52% of our patients, implying that approximately half of the cases are not caused by mutations in this gene, reinforcing the suggestion that the disease is genetically heterogeneous. Other studies have reported similar estimates for the prevalence of germline LKB1/STK11 mutations in PJS patients (Wang *et al*, 1999; Westerman *et al*, 1999b; Yoon *et al*, 2000; Olschwang *et al*, 2001). Some mutations may have gone undetected such as those in regulatory elements which may be undetectable in some PCR-based assays; however, families with PJS unlinked to 19p13.3 have been reported confirming that the disease is heterogeneous (Mehenni *et al*, 1998; Jiang *et al*, 1999; Westerman *et al*, 1999b; Yoon *et al*, 2000).

Studies that have formally estimated cancer risks in PJS have not computed separate estimates according to *LKB1/STK11* status. Olschwang *et al* (2001) recently reported a high frequency of proximal bile duct adenocarcinomas in PJS who did not carry *LKB1/STK11* mutations. Similarly, Boardman *et al* (2000) reported a high frequency of cancer in this group of patients, although no cases of bile duct cancers were observed. In our study, we had few familial cases not caused by *LKB1/STK11* mutations to enable us to compute a separate estimate of risk for noncarriers.

In conclusion, our results confirm that there is significant genetic heterogeneity in PJS. Future studies characterising the mutational status and disease manifestation in large numbers of PJS patients will allow better genotype–phenotype correlation to be made, which should assist clinicians in formulating cancer surveillance and individual predictive genetic testing.
